# Characterization and Validation of a Chronic Model of Cyclophosphamide-Induced Interstitial Cystitis/Bladder Pain Syndrome in Rats

**DOI:** 10.3389/fphar.2020.01305

**Published:** 2020-08-28

**Authors:** Céline Augé, Xavier Gamé, Nathalie Vergnolle, Philippe Lluel, Sophie Chabot

**Affiliations:** ^1^Department of Pain and Inflammation, Urosphere, Toulouse, France; ^2^Urology Department, Rangueil University Hospital, Toulouse, France; ^3^INSERM, I2MC-U1048, CHU Rangueil, Toulouse, France; ^4^IRSD, Université de Toulouse, INSERM, INRA, ENVT, UPS, Toulouse, France

**Keywords:** interstitial cystitis, bladder pain syndrome, cyclophosphamide, visceral pain, bladder inflammation, preclinical model

## Abstract

Interstitial cystitis/Bladder Pain Syndrome (IC/BPS) is a chronic inflammatory disease characterized by visceral pain and voiding symptoms. IC/BPS is still an unsolved enigma with ineffective diagnosis criteria and treatment. A main limitation in IC/BPS understanding is the lack of appropriate preclinical model. Cyclophosphamide (CYP) is commonly used as an experimental model for IC/BPS in rodent. However, the proposed models are very aggressive, contrasting with what occurs in clinic, and often associated with severe toxicity and high mortality rate. In addition, visceral pain, the hallmark symptom of IC/BPS, has been validated in only few of them. In this study, we developed a chronic model of CYP-induced IC/BPS in female rat. In our protocol, no severe weight loss occurred and the survival rate was 100%. In accordance to human pathology, chronic CYP-injected rats developed severe painful behavior whereas only sparse inflammation was observed. Inflammatory response was characterized by bladder edema and focal urothelial damage but absence of massive infiltrate. This chronic model showed persistent symptoms indicative for a central sensitization mechanism. We further demonstrate that CYP-induced chronic visceral pain was significantly reduced by curative treatment with clinically relevant compounds (gabapentin, ibuprofen, and Ialuril^®^). We therefore developed and validated a rat model of chronic cystitis that shares strong similarity with human non-ulcerative IC/BPS features without overtly affecting the animal health. This model will thus provide mechanistic insights of the disease and help to evaluate therapeutic agents for IC/BPS.

## Introduction

Interstitial cystitis/bladder pain syndrome (IC/BPS) is a chronic disorder characterized by bladder inflammation, pelvic pain, and voiding symptoms such as increased urinary frequency and urgency (Hanno et al., 2015). The symptoms range from mild to severe, and intermittent to constant (Driscoll and Teichman, 2001). Epidemiology studies of IC/BPS are hampered by lack of a uniform definition and absence of validated diagnostic marker. Estimation varies between 0.2% and 3.4% of the population, mostly affecting women (90%). This pathology has a profound impact on quality of life and can result in anxiety, sexual difficulties, loss of social interactions, and depression (Hepner et al., 2012). Throughout the last century, IC has been discovered, defined, and redefined innumerable times. Recently, IC/BPS was subcategorized into Hunner lesion interstitial cystitis (HL IC) and non-Hunner lesion interstitial cystitis and bladder pain syndrome (N-HL IC/BPS) (Han et al., 2018). The key difference between HL IC and N-HL IC/BPS is the presence of ulcerative Hunner lesions on cystoscopy. HL IC is relatively rare, with prevalence ranging from 5% to 20% whereas the N-HL IC/BPS is the phenotype more represented.

Although the aetiology and pathogenesis of IC/BPS have not yet been elucidated, numerous theories including defects of the urothelial barrier, autoimmunity and neurogenic disorder have been proposed (Parsons, 2007; van de Merwe, 2007). The aetiology of IC/BPS is likely to be multifactorial.

IC/BPS is still a challenging condition to treat. To date, two treatments are approved by the United States Food and Drug Administration (FDA). One is oral pentosan polysulfate (Elmiron^®^) and the other is intravesical dimethyl sulfoxide (DMSO). However, these two treatments are effective on limited number of patients and cause undesirable side effects (Hanno, 1997).

A wide variety of others treatments dedicated for pain management are used for IC/BPS. They include non-steroidal anti-inflammatory drugs (NSAIDs) (Kwon et al., 2013), the anticonvulsant gabapentin (Sasaki et al., 2001; Kwon et al., 2013), opioids (Wesselmann et al., 1997) or intravesical instillation of hyaluronic acid plus chondroitin sulfate such as Ialuril^®^ (Lazzeri et al., 2016) and (Cervigni et al., 2017). Nevertheless, most treatments demonstrate limited efficacy.

The inefficiency of IC/BPS therapies is mostly due to the poor understanding of the etiology and pathogenesis of the disease. Therefore, relevant preclinical models would be of great value to screen innovative drugs.

In humans, the chemotherapeutic drug cyclophosphamide (CYP) can induce hemorrhagic cystitis, increased voiding frequency and visceral pain. CYP-induced cystitis is believed to occur through accumulation of its toxic metabolic acrolein in the urinary bladder (Cox, 1979; Liu et al., 2010). Based on this clinical observation, CYP has been widely used in rodent as an experimental model of IC/BPS. Single CYP intraperitoneal injection leads to urinary bladder inflammation (Smaldone et al., 2009), visceral pain (Boucher et al., 2000) and detrusor overactivity (Juszczak et al., 2007). These typical features of IC/BPS appear within 4 h after CYP injection (Augé et al., 2013). However, in this acute model, massive local inflammation and tissue hemorrhage occurred making it more relevant to ulcerative form of IC, which has a prevalence of only 5% to 20% in IC/BPS patients (Parsons, 1990; Koziol et al., 1996).

To overcome these discrepancies, chronic models induced by multiple systemic injections of lower doses of CYP have been described in rats (Vizzard et al., 1996; Chopra et al., 2005; Arms et al., 2013). Nevertheless, they resulted in severe weight loss and caused up to 50% mortality (Vera et al., 2008a). Then, a less drastic rat chronic model has been offered (Vera et al., 2008a; Vera et al., 2008b). However, this model used male whereas IC/BPS affects mostly women. Furthermore, visceral pain was not evaluated although it is the main criteria for IC/BPS diagnosis.

In this study, we have developed and characterized a new model of chronic cystitis in rats in the context of its relevance to IC/BPS in human.

## Materials and Methods

### Animals

Sprague-Dawley female rats (7 weeks) were purchased from Janvier Labs (Le Genest Saint Isle, France). The animals were housed at a controlled temperature (21 ± 3°C) on a 12 h light/dark cycle with free access to food and water. Animals were acclimatized to the laboratory conditions for at least 3 days before the start of experiments. Animal protocols were approved by French Animal Ethical Committee (application number CEEA-122-2014-28 and APAFIS#16506-2018082411278474) and all procedures were conducted in accordance with the European Community Council Directive 2010/63/UE.

### Induction of Cystitis and Drug Treatments

**Figure 1:**
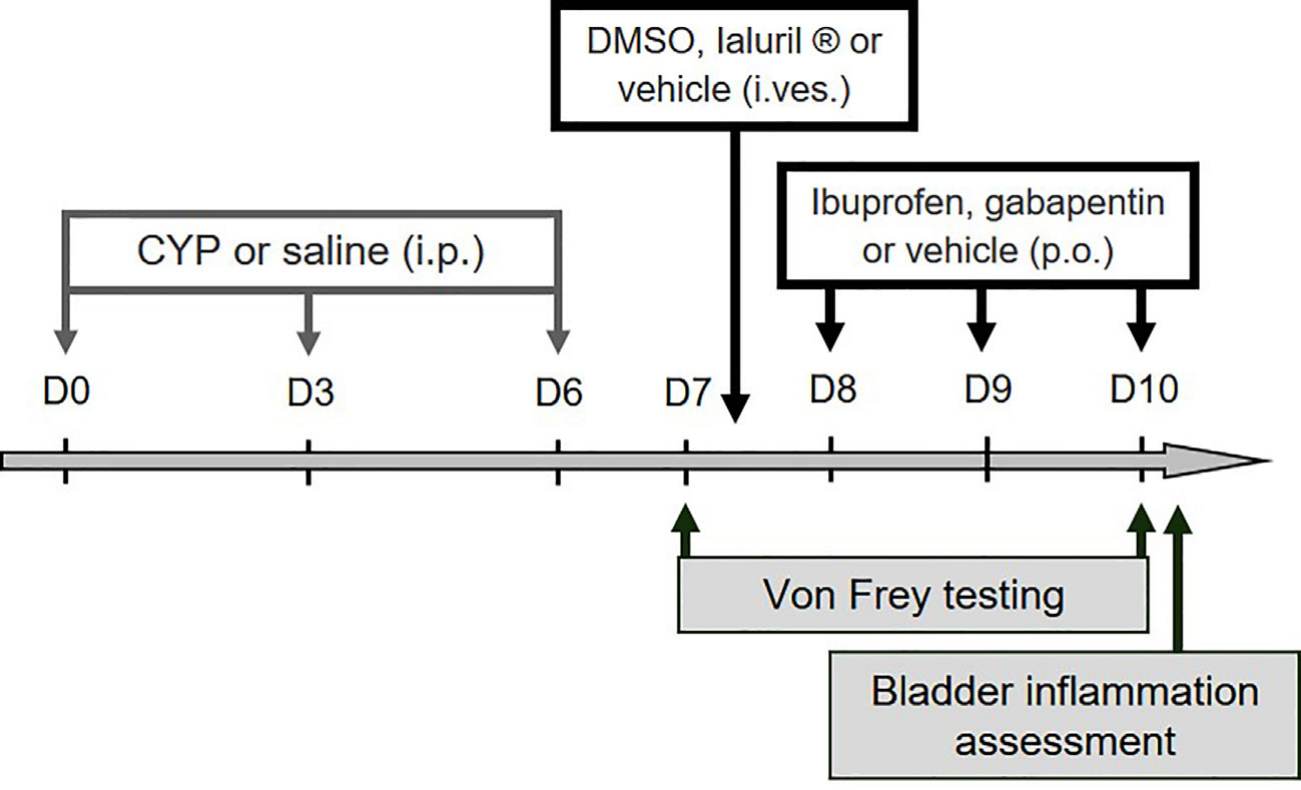
Experimental designs used in the study. For validation of the preclinical model, ibuprofen, gabapentin or vehicle was administered (100 mg/kg, p.o.) on days 8, 9, and 10. Intravesical instillation of DMSO (50%), Ialuril^®^ or vehicle (500 µL/rat) was performed under gaseous anesthesia on day 7. Von Frey test was first performed at day 7 (before pharmacological treatment) to confirm presence of visceral pain, and then at day 10 to analyze compounds effect on CYP-induced chronic visceral pain. Bladder inflammation was also assessed at D10 after von Frey test.

Reagents were purchased from Sigma-Aldrich (Saint-Louis, MO, USA) unless otherwise specified. Chronic cystitis was induced by one i.p. injection every 3^rd^ day (on days 0, 3, and 6) of CYP (Fisher-Scientific, Illkirch, France) at 40 or 75 mg/kg. Control animals received physiological saline (5 ml/kg, i.p) under the same experimental condition. Physiological saline (NaCl 0.9%) was purchased from VWR (Fontenay sous Bois, France). Gabapentin or ibuprofen (100 mg/kg) was administered orally on days 8, 9, and 10. Intravesical instillation of DMSO (50%) or Ialuril^®^ was performed under isoflurane anesthesia on day 7 (see the experimental design presented in **Figure 1**). Treatment (500 µl) was left in place for 30 min after which a gentle massage of the lower abdomen was performed to empty the bladder. Ialuril^®^ was purchased from IBSA *via* Pharmaclic (Gosselies, Belgium).

### Nociceptive Response to Mechanical Stimulation

Nociceptive response was evaluated, before CYP or saline injection (basal) and at indicated time after the first injection of CYP or saline. Eight von Frey filaments (Bioseb, Vitrolles, France) of increasing forces were applied to the lower abdomen, close to the urinary bladder as previously described (Augé et al., 2013). Standardized conditions for testing, including single-experimenter testing of all animals, were applied to minimize variability behavior-based pain testing. Prior testing, the abdominal area designed for mechanical stimulation of each animal was shaved. Animals were then placed on a raised wire mesh floor under individual transparent Plexiglas box and acclimatized for at least 30 min before starting the von Frey test. Filaments were then applied 1–2 s through the mesh floor with enough strength to cause the filament to slightly bend. Each filament was tested 3 times with an interval of 5 s between each application. Care was taken to stimulate different areas within the lower abdominal region in the vicinity of the urinary bladder to avoid desensitization. The scoring of nociceptive response was as follows: 0 = no response; 1 = reaction of the animal (*e.g.* retraction of the abdomen); 2 = reaction of the animal and change of position; 3 = reaction of the animal, change of position, licking of the area stimulated and/or vocalization.

Nociceptive score was expressed as the percentage of the maximal score for the three pooled applications.

### Bladder Inflammation Assessment and Histopathology

Animals were sacrificed at indicated times after the first injection of CYP or saline. Urinary bladders were rapidly collected and assessed for bladder weight, wall thickness and edema evaluation. Each bladder was macroscopically examined for edema and scored based on criteria established by Gray et al. (1986) as follow: absent (0), mild (1), moderate (2) and severe (3). Edema was considered severe when fluid was seen both externally and internally on the wall of the bladder. When edema was confined to the internal mucosa, it was reported as moderate. Finally, when edema was between normal to moderate, it was defined as mild.

Bladders were fixed in 10% formalin and embedded in paraffin. Bladder sections were stained with hematoxylin and eosin (HE) and digitized with a slide scanner (Nanozoomer, Hamamatsu, objective x20).

### Vesical Vascular Permeability

Vesical vascular permeability was evaluated by the Evans blue extravasation technique (Denadai-Souza et al., 2012). Ten days after the first CYP or saline injection, Evans blue (50 mg/kg) was intravenously injected 30 min before euthanasia. Blood samples were collected and animals were then perfused with saline to wash away intravascular dye. Urinary bladders were dissected and weighed. Tissue and plasma samples were incubated over 24 h at 60°C in formamide (1 ml/bladder or/10 µl of plasma). The concentration of extracted dye was determined by measuring the absorbance at 620 nm and by referring to that obtained with standard curve of Evans blue (1–50 µg/ml).

### Statistics

Quantitative data were analyzed with Prism 4 software (Graphpad, San-Diego, CA). Survival curve was computed using the Kaplan-Meier method. Log-rank test was applied to identify significant differences. For time-course experiments, two-way analysis of variance (ANOVA) with one between-subjects factor (*i.e.* treatment) and one within-subjects factor (*i.e.* time) was used to determine whether there was a significant main difference among groups. Two-way ANOVA was followed by *post hoc* Bonferonni’s multiple comparisons test to determine the basis of the significant main difference. When performing same measurement on a given animal at multiple time points, repeated measures (RM) were used. Differences between two means were assessed by t-test. Comparison of more than two means was made by a one-way ANOVA. Before performing statistical tests, we determined whether the data were normally distributed and evaluated their variance. We then conducted appropriate tests as indicated. Data were expressed as mean ± SEM. The accepted level of significance was p <0.05. The number of animal used per experiment is indicated in the legends.

## Results

### Dose Effect of Chronic Injection of CYP on Rat Body Weight and Survival

**Figure 2:**
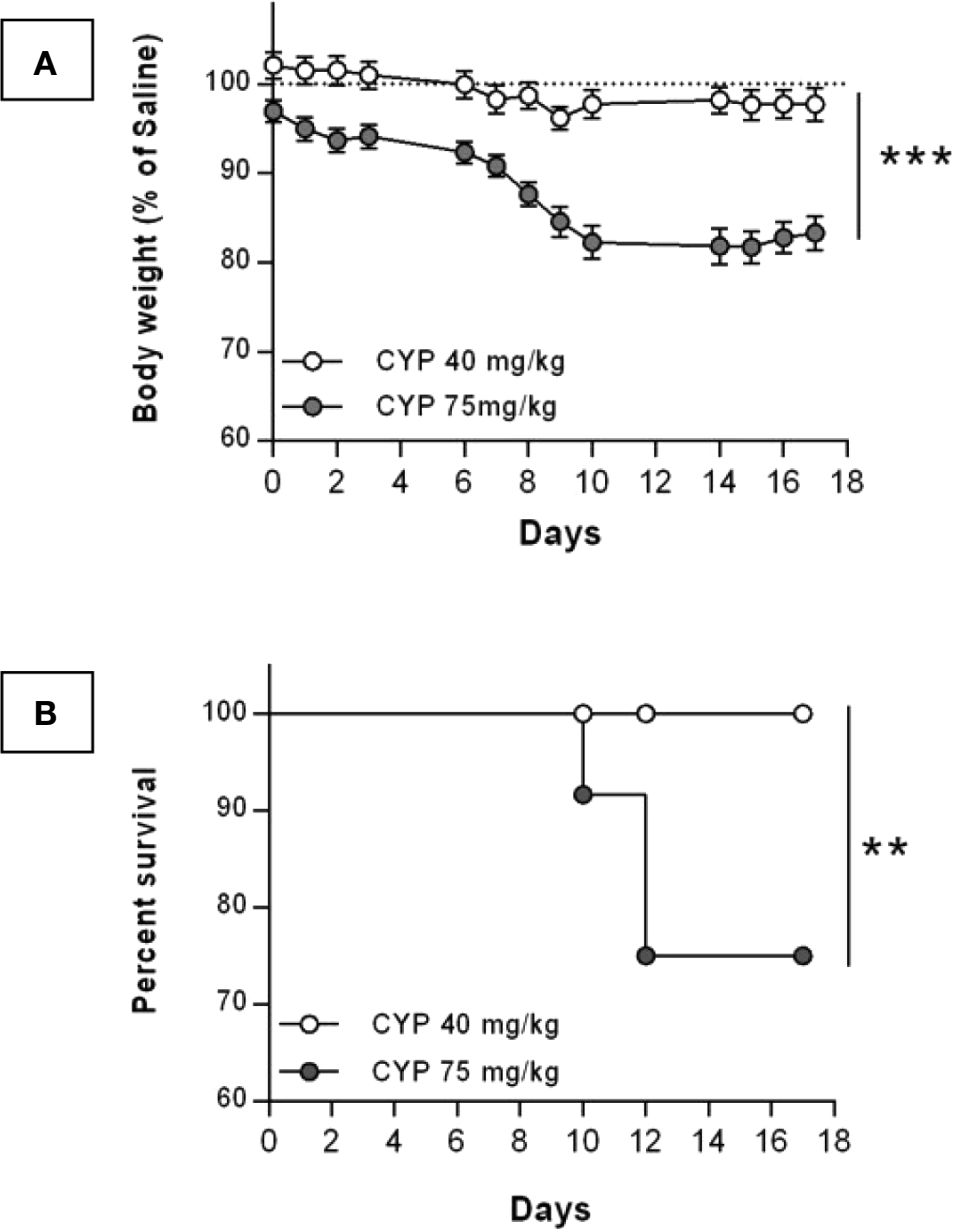
Change in body weight and survival after chronic injection of CYP at either 40 or 75 mg/kg dose. Rats received three intraperitoneal injections of saline (n = 12) at 5 ml/kg or CYP at either 40 (n = 24) or 75 (n = 12) mg/kg, on days 0, 3, and 6. **(A)** Body weight of CYP-injected rats were recorded over 17 days. Results were expressed as percentage of body weight of saline rats recorded at each time-point. Data represent mean values ± SEM. ***p < 0.001 (two-way RM ANOVA). **(B)** Kaplan-Meyer survival curves for rats chronically treated with either 40 or 75 mg/kg dose of CYP. **p = 0.0089 (Log-rank test).

For a chronic treatment, concerns about toxicity of CYP led us to test both the commonly published 75 mg/kg dose (Vizzard et al., 1996; Chopra et al., 2005; Arms et al., 2013) and the 40 mg/kg dose (Vera et al., 2008a; Vera et al., 2008b). CYP toxicity was indirectly assessed by recording rat body weight and mortality. The body weight of rats injected with CYP at 75 mg/kg was statistically lower compared to that of rats injected with the 40 mg/kg dose (**Figure 2A**). The 75 mg/kg dose produced severe weight loss and death. On the contrary, in the 40 mg/kg CYP group, only minor body weight decrease (<5%) was observed as compared to that of Saline group and the survival was 100% over 17 days (**Figures 2A, B**, respectively). Based on these results, we established our protocol with the 40 mg/kg dose of CYP administered once every 3^rd^ day (on days 0, 3, and 6).

### Chronic CYP Induced Persistent Visceral Pain and Inflammation

**Figure 3:**
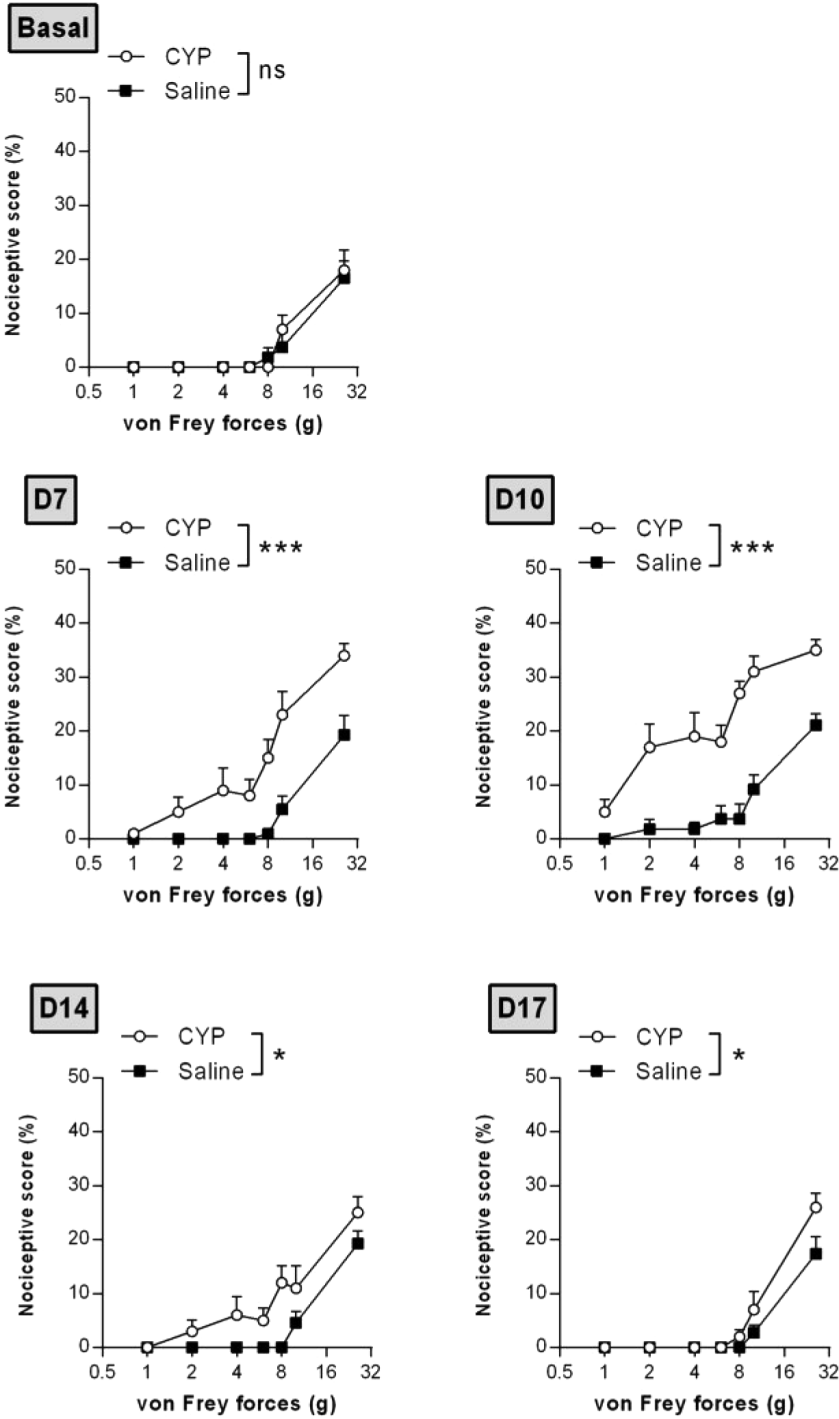
Repeated injections of low dose of CYP induced persistent visceral pain. Rats received three doses of CYP (40 mg/kg, i.p.) or saline (5 ml/kg, i.p.) on days 0, 3, and 6. Nociceptive response was assessed in a time-course study over 17 days by application of von Frey filaments of increasing force on the vicinity of the urinary bladder. Nociceptive score was expressed as the percentage of the maximal score for each von Frey force. Data represent mean values ± SEM (n =11 and 12 for CYP and Saline, respectively). ^ns^p > 0.05, *p < 0.05, ***p < 0.001 (two-way RM ANOVA).

**Figure 4:**
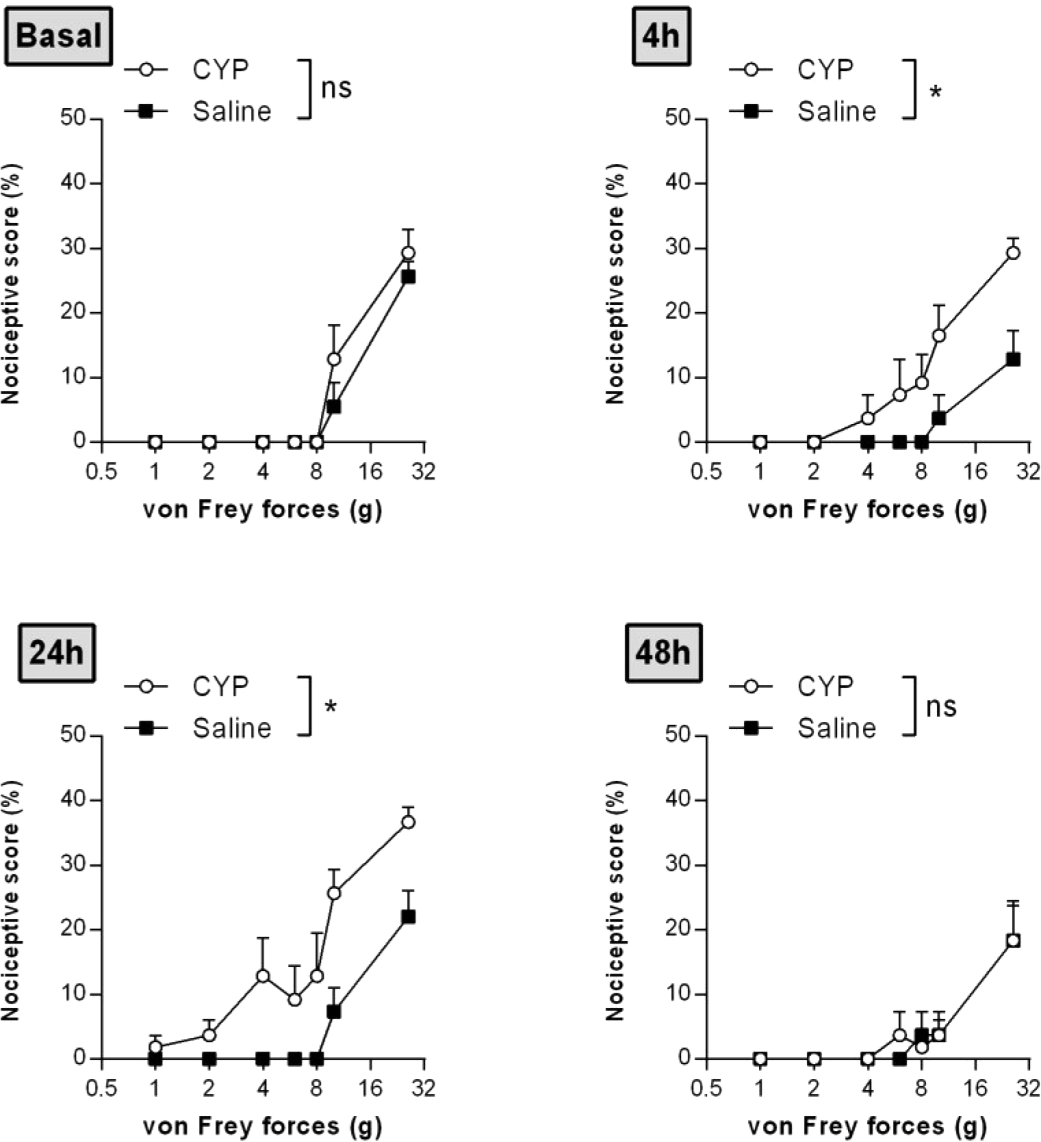
Single injection of CYP-induced short-lasting bladder pain. Rats received single dose of CYP (40 mg/kg, i.p.) or saline (5 ml/kg, i.p.). Nociceptive response was assessed in a time-course study over 48 h by von Frey testing. Nociceptive score was expressed as the percentage of the maximal score for each von Frey force. Data represent mean values ± SEM (n = 6/group). ^ns^p > 0.05, *p < 0.05 (two-way RM ANOVA).

Time course nociceptive response was evaluated by von Frey testing. **Figure 3** shows that chronic injection of CYP produced visceral pain which was characterized by a painful response to normally innocuous 1–8 g von Frey forces (allodynia) and by an increased response to a noxious 10–26 g von Frey forces (hyperalgesia). In chronic CYP-injected rats, visceral pain increased progressively reaching a peak at D10 (p < 0.001, **Figure 3**) after which CYP effect dropped to return closed to baseline at D17 (p < 0.05, **Figure 3**). Interestingly, single CYP injection at the same dose induced nociceptive response that lasted only until 24 h (**Figure 4**). All together, these results indicated that chronic visceral pain occurred in our model.

**Figure 5:**
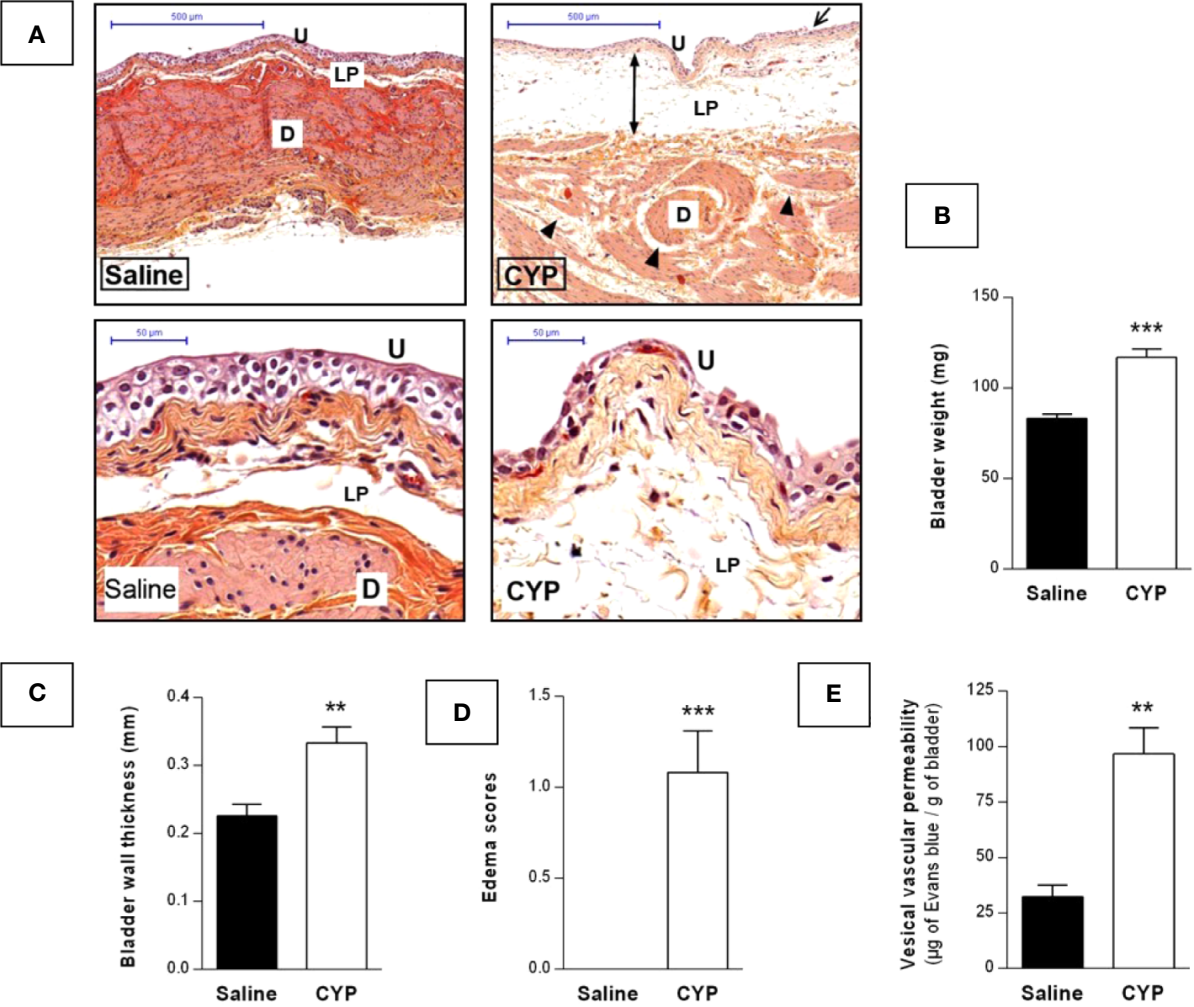
Repeated injections of low dose of CYP induced chronic bladder inflammation. Cystitis was induced as described previously and urinary bladders were collected at D10. **(A)** Representative H&E-stained sections of bladder from saline or CYP-injected rat. Edema in detrusor and lamina propria are indicated by arrowheads and injured urothelium is shown by arrows. Abbreviation used: D, detrusor; LP, lamina propria; U, urothelium. Scale bars, 500 µm (top images) and 50 µm (bottom images). Collected bladders were assessed for **(B)** weight, **(C)** wall thickness, and **(D)** edema scoring (n = 12/group). **(E)** Quantification of vesical vascular permeability assessed by Evans blue extravasation, 10 day after saline (n=5) or CYP (n=7) first injection. Data represent mean values ± SEM. **p < 0.01, ***p < 0.001 [**B**, unpaired t test with Welch’s correction; **(C–E)**, Mann Whitney test].

Bladder inflammation was then assessed at D10. Visual inspection of H&E-stained cross-sections showed that chronic CYP induced focal urothelial cell injury as indicated by cell swelling and urothelial thinning while only sparse inflammatory infiltrate was observed (**Figure 5A**). In agreement, no myeloperoxidase activity was detected in bladder tissue (data not shown). The main histological feature was severe edema in the lamina propria. We further showed that chronic CYP induced bladder weight and wall thickness increases as compared to saline (p < 0.001, **Figure 5B** and p < 0.01, **Figure 5C**). Edema score was also enhanced confirming our histological findings (p < 0.001, **Figure 5D**). Finally, we observed by Evans blue extravasation technique that CYP significantly increased vesical vascular permeability (p < 0.01, **Figure 5E**).

### Validation of the Model by Curative Treatment With Clinically Relevant Compounds

**Figure 6:**
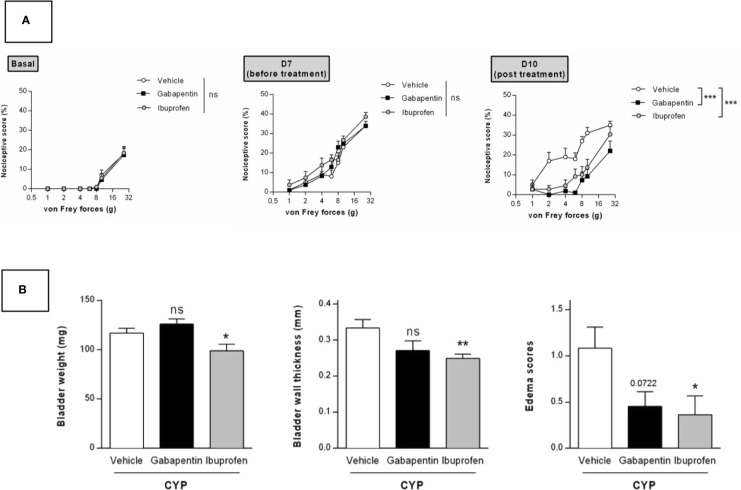
Validation of the model by curative oral treatment of clinically relevant compounds. Rats received three injections of CYP (40 mg/kg, i.p.) on days 0, 3, and 6. Rats were randomized and treated with three oral administrations of either ibuprofen (100 mg/kg) or gabapentin (100 mg/kg) or vehicle (10 ml/kg) on days 8, 9, and 10. **(A)** Nociceptive response was assessed by von Frey testing at day 7, once the cystitis was established but before treatment start (Before), and at day 10, 1 h after the last treatment (Post). Nociceptive score was expressed as the percentage of the maximal score for each von Frey force. **(B)** Bladder inflammation was assessed at day 10 by measuring bladder weight, wall thickness and edema scores. Data represent mean values ± SEM (n = 11 for CYP and n = 12 for Gabapentin and Ibuprofen). ^ns^p > 0.05, *p < 0.05, **p < 0.01, ***p < 0.001 (two-way RM ANOVA). **(A)** Two-way RM ANOVA; **(B)** Mann Whitney or unpaired t test).

We tested in our chronic model the effect of curative treatment of clinically relevant compounds. We first evaluated ibuprofen and gabapentin oral treatment (100 mg/kg, three administrations once a day, D8, D9, and D10, **Figure 1**). Von Frey test was performed before chronic cystitis induction (basal) and then after induction but prior pharmacological treatment (D7). No significant difference was observed among groups before treatment (D7) indicating that cystitis occurred similarly in all groups (p > 0.05, **Figure 6A**). Curative treatments with gabapentin or ibuprofen significantly decreased CYP-induced chronic visceral pain as compared to vehicle (p < 0.001, **Figure 6A**). Ibuprofen also significantly decreased bladder inflammation induced by CYP (p < 0.05, **Figure 6B**) while gabapentin was ineffective (p > 0.05, **Figure 6B**).

**Figure 7:**
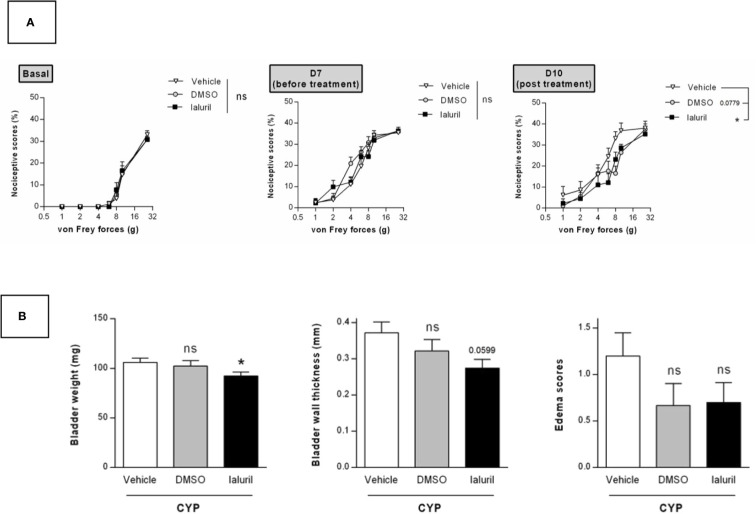
Validation of the model by curative intravesical treatment of clinically relevant compounds. Rats were treated with either DMSO (50%, i.ves.), Ialuril^®^ (i.ves.) or saline vehicle (500 µl/rat, i.ves.) on day 7. **(A)** Nociceptive response was assessed at day 7, once the cystitis was established but before start of the pharmacological treatment (Before) and at day 10 (Post). Nociceptive score was expressed as the percentage of the maximal score for each von Frey force. **(B)** Bladder inflammation was assessed at day 10 by measuring bladder weight, wall thickness and edema scores. Data represent mean values ± SEM (n = 10 for Vehicle and Ialuril^®^ and n = 9 for DMSO). ^ns^p > 0.05, *p < 0.05 **(A)** two-way RM ANOVA; **(B)** Mann Whitney or unpaired t test).

We then evaluated the effects of a single intravesical instillation of Ialuril^®^ and DMSO (50%). Instillation was performed after cystitis establishment at D7 (**Figure 1**). As above, von Frey test performed just before instillation indicated that magnitude of CYP effects was equivalent in all experimental groups (p > 0.05, **Figure 7A**). Ialuril^®^ but not DMSO displayed a significant but moderate inhibitory effect on CYP-induced chronic visceral pain (p < 0.05, **Figure 7A**). Results on visceral pain paralleled those obtained for bladder inflammation. In fact, slight decrease of bladder inflammation was observed after Ialuril^®^ with an effect that achieved the statistical significance level only for bladder weight (p < 0.05, **Figure 7B**) while DMSO had barely any effect (p > 0.05, **Figure 7B**).

## Discussion

IC/BPS is a pathology difficult to treat because the underlying etiology has not yet been elucidated. Complete diagnosis criteria is lacking and therapies are inefficient, so discovery of specific biomarkers and new pharmacological targets are needed. To achieve these goals, relevant animal models are a prerequisite.

Numerous preclinical models have been generated (Birder and Andersson, 2018) but the most widely used is based on a single systemic injection of CYP to induced acute pathology (Augé et al., 2013). Acute CYP causes severe bladder injury and massive inflammation indicating that this protocol is aggressive in comparison to clinical observation. Indeed, in non-ulcer IC, which has a prevalence of 80% to 95%, no or mild inflammatory infiltrate is observed (Harrington et al., 1990; Johansson and Fall, 1990). Moreover, IC/BPS is a chronic condition in which symptoms are persistent. In addition from more closely reproducing human disease, a chronic model allows curative treatment schedule which is clearly more relevant than a preventive treatment in terms of evaluation of new drugs. Thus, developing a chronic model that demonstrates all of the major phenotypical features of IC/BPS is of great value for the scientific community and pharmaceutical companies.

Chronic models have been described in mouse (Boudes et al., 2011; Golubeva et al., 2014). If mouse is of interest regarding the availability of large panel of genetically modified models, rat offers other advantages. In fact, rat is the model of choice for pharmacological studies and the transition to human is easier than for mice (Chiou and Barve, 1998; Iannaccone and Jacob, 2009; Aitman et al., 2016). Rat also allows repetitive biological sampling. It is noteworthy that rat is more sensitive to CYP as compared to mice, probably related to differential metabolism of CYP into acrolein (Simula and Priestly, 1992). In consequence, to adapt CYP mouse models to rats is challenging. It should be noted that in addition to specie specificity, strain differences in CYP sensitivity has also been reported (Bon et al., 2003). Strain dependence in CYP pain response is explained by difference in pharmacokinetics of CYP (Kanekal et al., 1992) but may be also due to a genetic influence on visceral nociceptive sensitivity (Bon et al., 2003). A lot of CYP rat studies were performed in Wistar rats (Vizzard et al., 1996; Hu et al., 2003) whereas Sprague-Dawley rats were used in this study. Thus, CYP dose must be also adjusted depending on the strain used.

Accordingly, CYP chronic models published in rats lead to deleterious effects such as strong body weight loss associated to high mortality, and severe bladder hemorrhage, which are atypical for non-ulcerative IC/BPS (Hu et al., 2003; Vera et al., 2008b). Vera et al. proposed a chronic model using lower CYP doses that showing no deleterious effect on animal (Vera et al., 2008a; Vera et al., 2008b). In their protocol, they used male rat whereas in clinic women are between five to ten times more likely to have cystitis than men (Moutzouris and Falagas, 2009). It is well known that gender affects CYP effect in rodents (Rodó et al., 2001; Rudick et al., 2012). Using female rats, we showed that the commonly published protocol for chronic CYP-induced cystitis, *i.e.* 75 mg/kg every 3^rd^ day elicited severe body weight loss leading to animal euthanasia in accordance to human endpoints (**Figure 2**). In contrast, the 40 mg/kg dose had no significant effect on body weight and survival rate was 100% (**Figure 2**).

Although visceral pain is the predominant characteristic of IC/BPS and is central to its diagnosis (Abrams et al., 2003), only one study, among those using rat CYP chronic model, addressed visceral pain (Meotti et al., 2013). However, this study used a too drastic protocol, *i.e.* CYP at 100 mg/kg. In the present study, we performed a time-course experiment to assess CYP-induced visceral pain. We showed that rats developed persistent painful behavior. CYP-induced visceral pain lasted until D10 (4 days after the last CYP injection) (**Figure 3**), whereas in acute CYP at the same dose, pain almost disappeared after 1 day (**Figure 4**). Detailed analysis of the clinical data suggests that IC/BPS is associated with changes in the central nervous system (CNS) (Ness et al., 2005; Kilpatrick et al., 2010; Woolf, 2011; Lai et al., 2014). Central sensitization may play a role in the maintenance of BPS symptoms. Taken together, these data prompted us to propose that repeated CYP injections over time resulted in enhanced responsiveness probably through central sensitization, reinforcing the value of chronic preclinical model in comparison to acute ones.

Central sensitization may also magnify perception so that non-painful stimuli are perceived as painful (allodynia) and painful stimuli become more painful than expected (hyperalgesia) (Fall et al., 2010). In fact, IC/BPS patients display allodynia during bladder distension and visceral hyperalgesia (Fitzgerald et al., 2005; Giamberardino et al., 2014; Ness et al., 2014). Accordingly, we found that CYP-induced chronic visceral pain was characterized by both allodynia and hyperalgesia.

We also focused on inflammatory response occurring in our model. CYP-induced inflammation was characterized by increased bladder weight, wall thickness as well as edema scores (**Figure 5**) and by histological profile including lamina propria edema and focal urothelial damage. Interestingly, little or no inflammatory cells infiltration was observed (**Figure 5**). These features are similar to those of human IC/BPS. Indeed, there are now numerous clinical data showing no or sparse inflammatory infiltrate in non-ulcerative IC/BPS patient (Messing and Stamey, 1978; Harrington et al., 1990; Johansson and Fall, 1990; Logadottir et al., 2014). Moreover, submucosal edema and discohesive urothelium are characteristic histological findings in IC/BPS patients (Messing and Stamey, 1978; Johansson and Fall, 1990).

Finally, we pharmacologically validated our model by using clinically relevant drugs. We thus demonstrated that a curative oral treatment with either ibuprofen or the anticonvulsant gabapentin decreased CYP-induced chronic visceral pain (**Figure 6**). Gabapentin is a structural analogue of γ-aminobutyric acid which has been effectively used in various chronic pain conditions such as IC/BPS (Lee et al., 2010; Kwon et al., 2013). We also showed that ibuprofen decreased CYP-induced bladder inflammation (**Figure 6**). Conversely, although gabapentin completely abolished referred visceral pain, it had no effect on bladder inflammation (**Figure 6**). This result suggests that both inflammatory and pain symptoms must be considered when designing therapy. Thus, combined therapy with NSAIDs and pain killers may represent a promising therapeutic strategy (Lee et al., 2010; Kwon et al., 2013).

Currently, one of the strategy of choice in IC/BPS is intravesical treatment. Various intravesical therapies are used for IC/BPS, including heparin, hyaluronic acid, chondroitin sulfate, pentosan polysulfate, DMSO, liposomes, and botulinum onabotulinumtoxinA *(*Lazzeri et al., 2016*;* Meng et al., 2018). Since 1978, 50% DMSO is marketed as (Rimso-50) as an intravesical treatment of IC/BPS (Shirley et al., 1978). Beneficial effects of DMSO are controversial and the European Association of Urology guidelines recent update does not recommend the use of DMSO (Engeler et al., 2018). Ialuril^®^ is an association of sodium hyaluronate, chondroitin sulphate and calcium chloride. It was designed to facilitate faster and more effective restoration of the bladder epithelium. Instillation regimen is repeated instillations over 5 months (Cicione et al., 2014). Numerous studies were conducted using combination of hyaluronic acid and chondroitin sulphate but there were differences in proven efficacy. In our model, we observed that Ialuril^®^ had a moderate but significant effect on CYP-induced chronic visceral pain whereas DSMO had barely any effect (**Figure 7**). These last results showed that our model can be of interest for intravesical-based therapy evaluation.

As concluded in the International Consultation on Incontinence-Research Society meeting; “translational research studies are still in need of improved animal models to study IC/BPS mechanisms and development of novel methods to objectively measure bladder pain in rodents” (Malykhina and Hanno, 2014). In the present study, we developed a new preclinical chronic model of IC/BPS with persistent symptoms that recapitulates the key features of human non-ulcerative IC/BPS, which accounts for more than 80% of IC patients (Parsons, 1990; Koziol et al., 1996). These included sustained visceral pain and mild inflammatory response in bladder tissue characterized by edema in the lamina propria, focal urothelial injury and absence of massive infiltrate or tissue hemorrhage. This model is of significant value for better understanding pathophysiological mechanisms and helping finding new therapies for IC/BPS.

## Data Availability Statement

The raw data supporting the conclusions of this article will be made available by the authors, without undue reservation, to any qualified researcher.

## Ethics Statement

The animal study was reviewed and approved by French Animal Ethical Committee.

## Author Contributions

CA performed and planned the experiments. CA and SC designed and analyzed the data. SC with support of CA wrote the manuscript. XG. NV, and PL discussed results and contributed to the final manuscript. SC supervised the study and PL directed the project.

## Funding

This work was part of the “MAGenTA” Program overseen by “La Banque Publique d’Investissement (BPI)” given to Urosphere for implementation of experimental model for urogenital tract pathologies.

## Conflict of Interest

The authors declare that the research was conducted in the absence of any commercial or financial relationships that could be construed as a potential conflict of interest.
